# Role of artificial intelligence and machine learning in the diagnosis of cerebrovascular disease

**DOI:** 10.3389/fnhum.2023.1254417

**Published:** 2023-09-07

**Authors:** Kevin Gilotra, Sujith Swarna, Racheed Mani, Jade Basem, Reza Dashti

**Affiliations:** Dashti Lab, Department of Neurological Surgery, Stony Brook University Hospital, Stony Brook, NY, United States

**Keywords:** artificial intelligence, machine learning, deep learning, cerebrovascular, ischemic stroke, hemorrhagic stroke, aneurysm, arteriovenous malformation

## Abstract

**Introduction:**

Cerebrovascular diseases are known to cause significant morbidity and mortality to the general population. In patients with cerebrovascular disease, prompt clinical evaluation and radiographic interpretation are both essential in optimizing clinical management and in triaging patients for critical and potentially life-saving neurosurgical interventions. With recent advancements in the domains of artificial intelligence (AI) and machine learning (ML), many AI and ML algorithms have been developed to further optimize the diagnosis and subsequent management of cerebrovascular disease. Despite such advances, further studies are needed to substantively evaluate both the diagnostic accuracy and feasibility of these techniques for their application in clinical practice. This review aims to analyze the current use of AI and MI algorithms in the diagnosis of, and clinical decision making for cerebrovascular disease, and to discuss both the feasibility and future applications of utilizing such algorithms.

**Methods:**

We review the use of AI and ML algorithms to assist clinicians in the diagnosis and management of ischemic stroke, hemorrhagic stroke, intracranial aneurysms, and arteriovenous malformations (AVMs). After identifying the most widely used algorithms, we provide a detailed analysis of the accuracy and effectiveness of these algorithms in practice.

**Results:**

The incorporation of AI and ML algorithms for cerebrovascular patients has demonstrated improvements in time to detection of intracranial pathologies such as intracerebral hemorrhage (ICH) and infarcts. For ischemic and hemorrhagic strokes, commercial AI software platforms such as RapidAI and *Viz.*AI have bene implemented into routine clinical practice at many stroke centers to expedite the detection of infarcts and ICH, respectively. Such algorithms and neural networks have also been analyzed for use in prognostication for such cerebrovascular pathologies. These include predicting outcomes for ischemic stroke patients, hematoma expansion, risk of aneurysm rupture, bleeding of AVMs, and in predicting outcomes following interventions such as risk of occlusion for various endovascular devices. Preliminary analyses have yielded promising sensitivities when AI and ML are used in concert with imaging modalities and a multidisciplinary team of health care providers.

**Conclusion:**

The implementation of AI and ML algorithms to supplement clinical practice has conferred a high degree of accuracy, efficiency, and expedited detection in the clinical and radiographic evaluation and management of ischemic and hemorrhagic strokes, AVMs, and aneurysms. Such algorithms have been explored for further purposes of prognostication for these conditions, with promising preliminary results. Further studies should evaluate the longitudinal implementation of such techniques into hospital networks and residency programs to supplement clinical practice, and the extent to which these techniques improve patient care and clinical outcomes in the long-term.

## 1. Introduction

Cerebrovascular disease encompasses a wide range of pathologies that can confer a high risk of potentially life-threatening sequelae; hence, timely diagnosis and treatment is essential in preventing subsequent severe neurological deterioration (Santana Baskar et al., 2021). This requires a large team of clinicians and support staff to effectively work up and manage these patients, to enable them to receive the highest quality of care. On initial presentation outside of the hospital, emergency medical staff must quickly recognize symptoms of cerebrovascular disease, safely transfer the patient to the hospital, and obtain stroke imaging as early as possible (Santana Baskar et al., 2021). This is in tandem with timely clinical evaluation immediately on admission to determine the patient’s neurological status and overall clinical picture, while also determining medical management, such as whether a patient is a candidate for medical thrombolytic therapy even before further intervention (Cumbler, 2015). From there, the radiology technologists and neuroradiologists work together to capture and interpret the appropriate imaging from which clinicians can hone in both on the critical diagnosis and in decision making for intervention/s (Cumbler, 2015; Green et al., 2021).

In recent years, with advances in technology and advanced machinery, health care has been incrementally augmented by the use of such software and technology to aid in diagnosis and decision-making for various medical conditions (Shuaib et al., 2020). Artificial intelligence (AI) is actively being implemented into many fields in medicine, and recent advancements in AI algorithms and machinery for diagnosing and treating cerebrovascular disease have the potential to revolutionize patient care (Soun et al., 2021). In order for AI to be further incorporated into the standard of care for cerebrovascular disease patients, many years of active collaboration between AI algorithm engineers and physicians are needed. In light of the current AI revolution in medicine, it is increasingly essential for health care professionals treating cerebrovascular patients to familiarize themselves with the applications of these innovations to their own field. By enhancing their knowledge, clinicians will find themselves more prepared when AI inevitably becomes an inherent part of future clinical practice (Lanzagorta-Ortega et al., 2022). In this review, we seek to provide an overview and evaluation of AI technologies applied to the field of cerebrovascular disease in the diagnosis of primary and secondary (lesional) hemorrhagic stroke, and ischemic stroke (IS). We performed a generalized review of the current literature by identifying articles that assessed the most updated AI and ML techniques. This was done by selecting the most relevant articles in the current cerebrovascular disease literature cited on the PubMed and Web of Science databases. In doing so, we aimed to summarize the clinical relevance of AI in cerebrovascular disease in simple terms so that practicing clinicians can gain a better appreciation of its current and potential future applications for cerebrovascular patients.

### 1.1. Terminology

Since this review requires clinicians to have a reasonable understanding of the fundamental concepts of AI, a discussion of relevant terminology would be beneficial.

**Figure 1:**
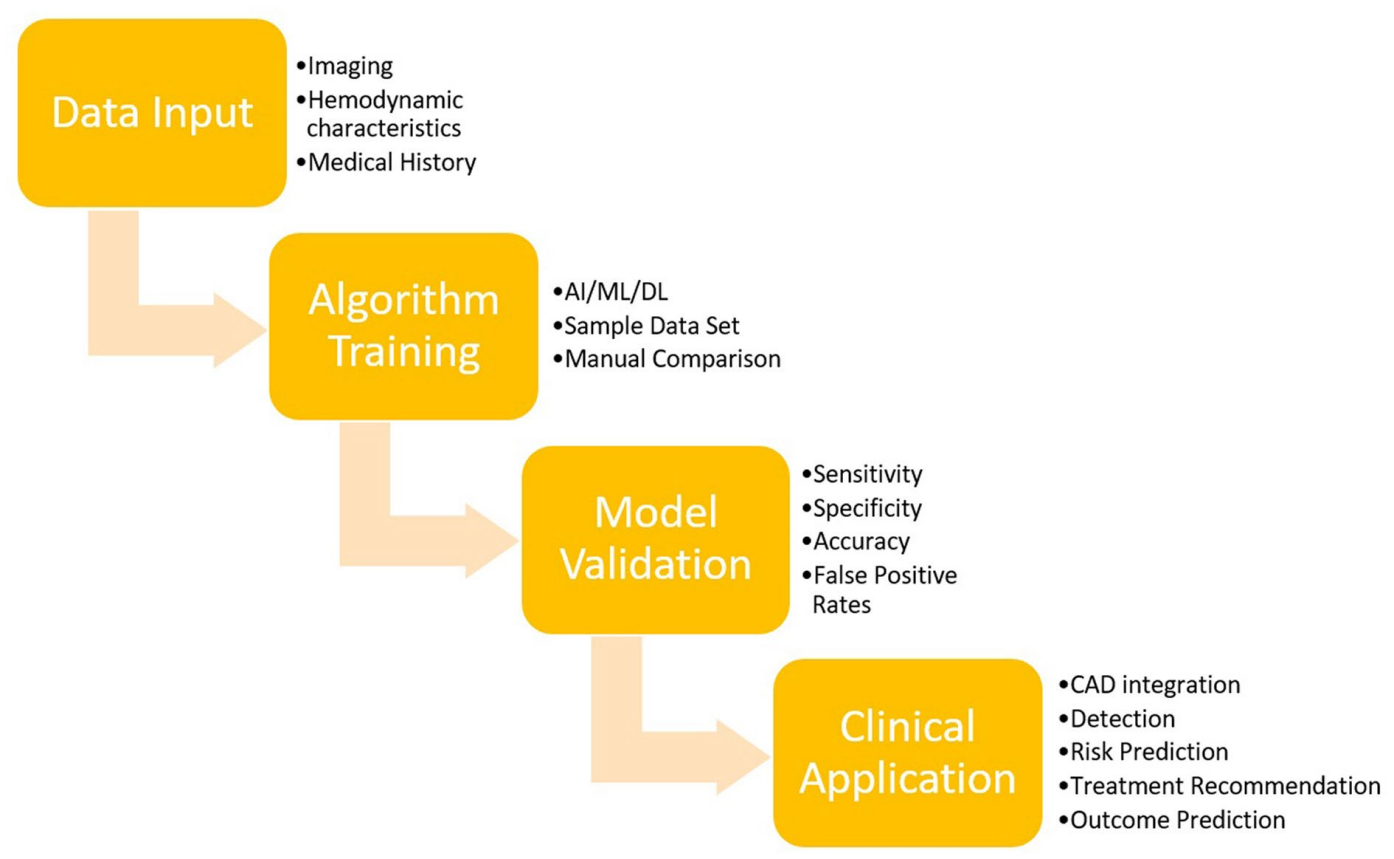
Overview of AI training and validation.

AI fundamentally refers to the ability of a machine to solve tasks in a way that simulates human intelligence (Moor, 2006). Machine learning (ML), a subset of AI, utilizes large data sets to train computers to iteratively generate a model based on recognizing rules or patterns in data (Choi et al., 2020). After training, the model is then tested with real-life data (testing data) to assess its accuracy. A schematic of this process is shown in Figure 1. In ischemic stroke, ML algorithms can be developed to demonstrate the presence of infarct, the total area of infarcted brain tissue, and the occluded vessel in question. Such an algorithm may subsequently be tested on a group of non-contrast computer tomography scans (NCCTs), with results then compared to the interpretation of the NCCTs by a radiologist to ascertain the accuracy of the algorithm (Wang and Summers, 2012; Choi et al., 2020; Kim et al., 2023).

Models are often trained using three major classifications of learning: supervised, unsupervised, and reinforcement (Sarker, 2021). In supervised learning, the training data consists of pre-labeled information. With unsupervised learning, pre-existing information is absent which means the model must cluster a group of cases together based on similar characteristics and identify the relationships between said groups (Sarker, 2021). In contrast to unsupervised learning, reinforcement learning allows the model to analyze pre-existing data and determine the “correct” and “incorrect” answers for any given scenario.

**Figure 2:**
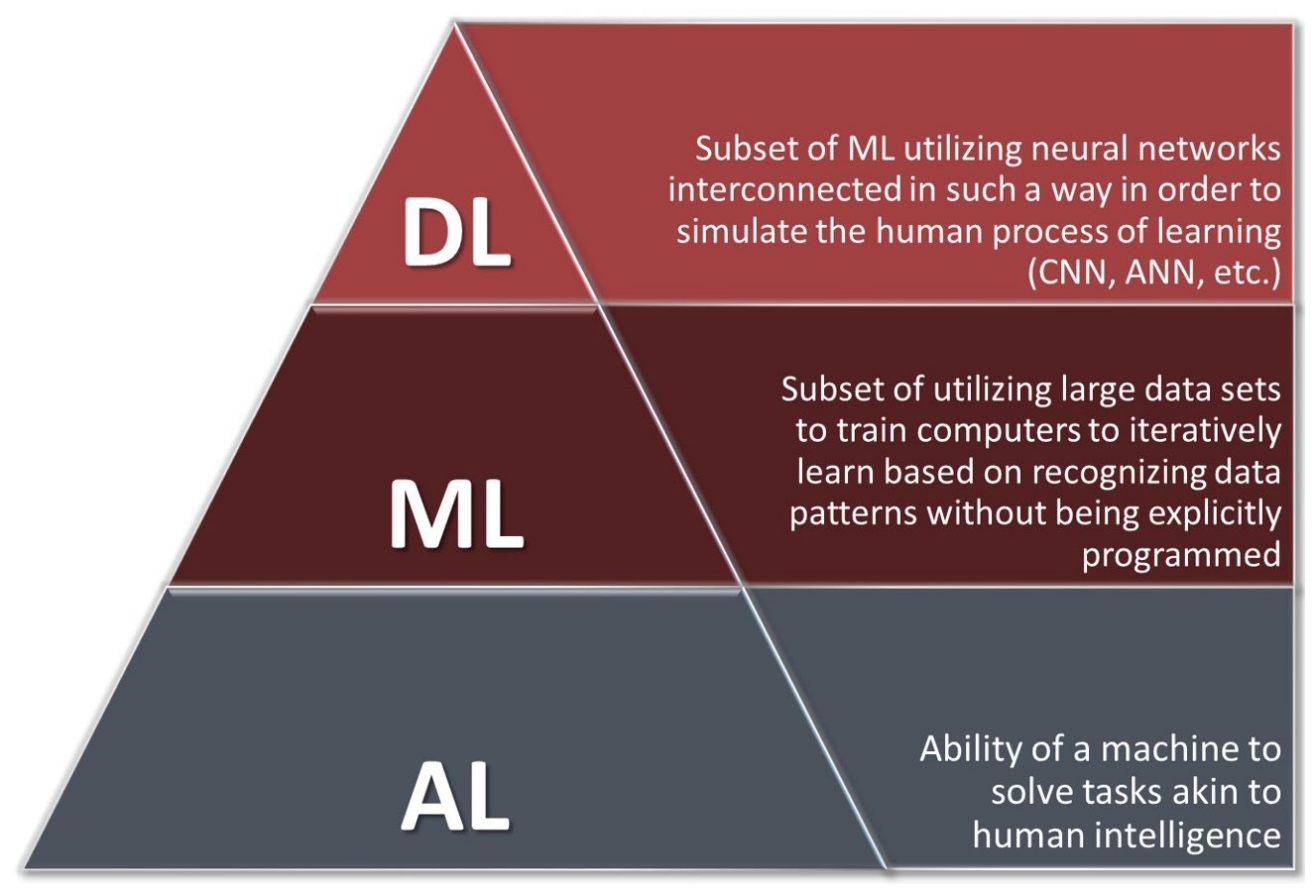
Schematic conceptual representation of AI, ML, and DL.

Deep learning (DL), a further subclassification of ML, utilizes neural networks, a series of nodes or layers that are interconnected in such a way in order to simulate the human process of learning (Chen et al., 2022). The network begins with an input layer and ends with an output layer. In between both of these layers are a series of hidden layers, each with a given weight and bias (Jovel and Greiner, 2021). Each layer receives the input and assigns a weight to it. When the output exceeds a given bias/threshold, an activation function is applied and then fed forward to the next node. During the training phase, the model can adjust these weights and biases accordingly until it achieves the desired output (Jovel and Greiner, 2021). One such application may include software that performs a rapid analysis of a NCCT for a patient with acute ICH and then decides whether or not the neuroradiologist should immediately be notified to interpret the film or if the neurosurgery team should be notified to prepare an operating room. Ultimately, three major classifications of deep learning exist and are characterized primarily by the kind of data they use optimally. Artificial neural networks (ANNs) work best with numeric input, convolutional neural networks (CNNs) with visual input, and recurrent neural networks (RNNs) with time-series data (Banerjee et al., 2019). These concepts are outlined schematically in Figure 2.

## 2. Hemorrhagic stroke

### 2.1. Background

Hemorrhagic stroke, also referred to as intracerebral hemorrhage (ICH), has multiple subtypes based on anatomical location of the bleed, encompassing subarachnoid hemorrhage (SAH), intraventricular hemorrhage (IVH), intraparenchymal hemorrhage (IPH), epidural hematoma (EDH), and subdural hematoma (SDH) (Soun et al., 2021). ICH carries a significant morbidity and mortality that is steadily increasing in prevalence worldwide, with fatality ranging anywhere from 30 to 65% of all incidents (Rymer, 2011; Vangen-Lønne et al., 2017). Therefore, a timely diagnosis and neurosurgical intervention (if warranted), are essential for ICH patients to improve clinical outcomes (Forman et al., 2020). This is of the utmost importance when neuroimaging suggests the presence of hematoma expansion (HE), defined as an increase in ICH volume by greater than 33% from the initial collection of hemorrhage (Forman et al., 2020; Wang et al., 2021). The diagnosis of ICH can often be made through an initial NCCT of the head. Characterization of the ICH is subsequently performed through CT Angiograms (CTA), Computed Tomography Perfusion (CTP), Digital Subtraction Angiography (DSA), and Magnetic Resonance Imaging (MRI) to ultimately guide further management (Forman et al., 2020).

Neuroradiologists, neurosurgeons, and neurologists receive years of training to accurately diagnose ICH subtypes and to obtain volume measurements using the above imaging modalities. However, in the modern era, newly developed AI and ML algorithms can assist physicians to identify ICH and HE using standard imaging techniques (Wang et al., 2021). The utilization of these algorithms can both ease the interpretation of imaging and more accurately quantify hematoma volumes to improve diagnostic accuracy and guide decision-making as it pertains to acute surgical intervention or conservative and medical management (Guo et al., 2022). Many of these management decisions are dictated by whether the patient is likely to have a poor prognosis in the long-term.

HE is a pathological feature present in up to 40% of ICH patients, and is known to be one of many prognostic indicators of poor outcomes (Qureshi and Palesch, 2011). This makes it essential to accurately quantify hematoma volumes at admission and in serial imaging for ICH patients (Helal et al., 2019; Li et al., 2020). Prior history of anticoagulant/antiplatelet usage, higher baseline ICH volumes and lower admission GCS scores are the most notable risk factors for developing HE (Guo et al., 2022). Furthermore, patients with HE subsequently are at greater risk of developing IVH and hydrocephalus (Yaghi et al., 2014). Despite the extensive literature surrounding ICH outcomes, HE is one of the few markers of outcome that can specifically be mitigated, and in some cases, prevented by neurosurgical intervention (Brouwers and Greenberg, 2013). This makes the identification of HE crucial during the care of ICH patients, as it effectively guides medical and surgical therapy while potentially preventing adverse outcomes (Aziz et al., 2015). The role of AI in these contexts pertains specifically in diagnosing subtypes of ICH, measuring ICH volumes, and in identifying HE and predictive signs of poor outcomes. Below, we discuss the current role of AI as well as how future technology can be implemented to improve neuroradiological care for ICH patients.

### 2.2. Standard techniques

One of the first techniques developed to evaluate ICH and measure hematoma volumes is manual segmentation (Nguyen et al., 2020). This entails the manual review of patients’ head CTs by individual slices to calculate the hematoma volume (HV). Although manual segmentation is the gold standard per the current literature, given its high accuracy and error-proof methodology, it is often time consuming, particularly in ICH patients for whom timely diagnosis is essential. Moreover, with the increasing need for imaging in a growing population of ICH patients, efficient review of CT scans and other imaging modalities is critical. With this rising demand, the ABC/2 technique was developed. With this technique, “A” is defined as the length of the longest layer diameter, “B” defined as the perpendicular vertical line to A, and “C” is a by-product of layer thickness multiplied by bleeding layer number. The approximate value of HV is then estimated as the product of these variables divided by two. Many studies have demonstrated limited efficacy of the ABC/2 method, citing both overestimation and underestimation of HVs and perihematomal edema (PHE) (Wang et al., 2009; Chen et al., 2022; Hillal et al., 2022). As a result, ABC/2 was often unreliable at many level one stroke centers (Webb et al., 2015).

Since larger HVs and PHE are arguably the most important predictors of HE and poor prognosis in acute ICH, accurate detection and quantification of these variables are essential (Yaghi et al., 2014; Li et al., 2020). With an aging population and increasing incidence rates of ICH, there has been a growing demand for institutions to develop AI and ML algorithms that accurately detect PHE and calculate HVs. A select few of the most promising algorithms are reviewed below.

### 2.3. ML for qualitative detection of ICH

With the growth of AI as a prospective tool for ICH detection and classification, there have been many studies which have utilized and evaluated some of the algorithms and neural networks outlined above. The primary outcome reported in the literature is area under the curve (AUC), which is a marker for the predictive accuracy of the model on a scale of 0–1. One of the highest-powered studies was a retrospective study examining over 30,000 CT scans across India using deep learning algorithms in two datasets (Qure25k and CQ500) (Chilamkurthy et al., 2018). The authors utilized a natural language processing (NLP) algorithm to detect IPH, SDH, EDH, SAH, and IVH, as well as other pertinent findings such as calvarial fractures, mass effect, and midline shift. The authors noted that both datasets demonstrated strong degrees of accuracy per the consensus of independent radiologists in detecting both the actual hemorrhages as well as the other pertinent CT findings above with an AUC of 0.92 and 0.94 for the above datasets. Another study from 2021 assessed 25,000 CT scans using more advanced two sequence models with 2D CNN to classify subtypes of ICH; they reported AUCs greater than 0.98 for SAH, IVH, SDH, EDH, and IPH (Wang et al., 2021). Nishi et al. (2021) elucidated an algorithm solely designed for SAH detection with NCCTs and compared its detection results with five neurosurgeons and five general practitioners (Nishi et al., 2021). Across 135 patients with SAH, their algorithm demonstrated similar performance to the neurosurgeons and stronger performance than four of the other five physicians (Nishi et al., 2021). The findings for all of these studies above suggest that AI is capable of classifying ICH subtypes by evaluating simple neuroanatomy at a level that is on par with well-trained physicians,

Other 2D-CNNs utilized in the evaluation of ICH include GoogLeNet and AlexNet, which have been applied to the detection of basal ganglia hemorrhage (Desai et al., 2017). Desai et al. noted that both CNNs demonstrated a high degree of accuracy in detecting deep ICH (Desai et al., 2017). GoogLeNet, with a pretrained network, yielded the highest accuracy (sensitivity and specificity of 100, AUC = 1.0). The untrained AlexNet yielded a high but slightly reduced level of accuracy compared to GoogLeNet (sensitivity = 100%, specificity 80%, AUC = 0.95) (Desai et al., 2017). These results demonstrate the capacity for such algorithms to provide significant diagnostic value even for deep ICH.

Arbabshirani et al. also evaluated a fully 3D-CNN to not only detect ICH, but also to effectively triage CT scans to prioritize radiology worklists and expedite diagnosis times (Arbabshirani et al., 2018). This network was trained on over 37,000 studies and prospectively evaluated on 9,499 hitherto unseen studies. This model was able to successfully re-prioritize 94 studies from “routine” to “stat,” and reduced the time to detection of ICH from 512 to 19 min. These findings demonstrate the further benefit not just in diagnosing ICH, but in expediting the process of diagnosis.

Another critical imaging modality in the detection of acute ICH is CTA. CTA allows clinicians to determine whether ICH is spontaneous, secondary to trauma, or from a pre-existing lesion such as a ruptured aneurysm or arteriovenous malformation (AVM) (Fu et al., 2023). Unlike NCCTs of the head which can be well-evaluated by most physicians, they require more subspecialist training to evaluate and often need more time to interpret in order to elicit an accurate diagnosis (Wada et al., 2007; Fu et al., 2023). One study evaluating a DL algorithm for 3,266 patients with CTAs of the head and neck showed a mean reduction time of 16.4 min when compared to trained radiologists with almost identical diagnostic accuracy (Fu et al., 2023). More research is needed to demonstrate the benefits of AI for interpretation of CTAs and other more complex imaging modalities such as MRIs/MRAs and DSAs in the context of ICH, as the current literature has primarily evaluated the use of AI in simple imaging techniques such as NCCTs (Hotta et al., 2014).

### 2.4. Novel machine learning techniques for quantitative hematoma evaluation

Most ML algorithms work by taking a pixel-wise approach and combining CT slice thickness to calculate HVs. Dhar et al. utilized a CNN model developed from U-Net architecture that demonstrated similar efficacy to manual segmentation techniques for calculating HVs and PHE (Dhar et al., 2020). Voxel-by-voxel overlap of hematoma segments was calculated using the Dice similarity coefficient (DSC), with a score of “0” suggesting no overlap at all and “1” suggesting maximum overlap between the manual segmentation method and the algorithm method. Across 224 CT scans in 124 patients, this study found that the UNet model had a DSC of 0.9 (IQR 0.85–0.93) for measuring HVs but only a DSC of 0.54 when measuring PHE. However, Ironside et al. (2020) used a fully automated segmentation algorithm with a goal of specifically calculating PHE in 400 patients with ICH. The automated algorithm was both faster and more accurate at detecting PHE (mean 480.5 ± 295.3 s/scan; *p* < 0.0001) and manual (mean 316.4 ± 168.8 s/scan; *p* < 0.0001) methods (Ironside et al., 2020).

Although both studies demonstrate strong efficacy of the algorithms developed, the conflicting results highlight the discrepancies that may exist between different ML algorithms when it comes to calculating PHE. Ironside et al. have followed up on their previously published results with the prospective QUANTUM study in 2022. Currently underway, this study intends to address relevant design considerations for an AI to accurately evaluate PHE in ICH patients (Ironside et al., 2022). When neurosurgeons use AI to help interpret NCCTs with acute hematomas, it is crucial that they are aware of how the AI was initially designed. This allows neurosurgeons to combine the appropriate mental resources from their clinical training with the expertise of the AI machinery to make faster and more accurate interpretations. While few subsequent studies in the literature have followed up on the evaluation of PHE, several studies have demonstrated the efficacy of using ML algorithms to calculate HVs. Yu et al. utilized “DR-UNet,” a dimensional reduction analytical framework upgraded from the CNN UNet model used by Yu et al. (2022). Across 562 patients with a collective 13,825 CT scans, the DR-UNet model’s hematoma volume calculations demonstrated a strongly positive correlation with the calculations made by a neuroradiologist using manual segmentation (*R*^2^ = 0.9979, *p* < 0.0001). Few prior studies have demonstrated this level of statistical power with ML algorithms. The DR-UNet model also accurately evaluated 13 irregularly shaped hematomas that were shaped differently from the remainder of hematomas in the rest of the data set, thus demonstrating the potential for ML algorithms to be used even in evaluating atypical hematomas (Yu et al., 2022).

Such hematomas are often better evaluated using CTP imaging. CTP is an imaging technique that allows for the visualization and volumetric measurement of cortical infarcts well as adjacent penumbra. Wang et al. also developed an algorithm for CTP images that evaluated 49 ICH patients with concurrent interventricular hemorrhage (IVH), a feature known to be associated with poor outcomes in ICH patients (Wang et al., 2021). They found no statistically significant difference between CTP-based planimetry segmentation and their algorithm (*p* = 0.614), despite strong correlations between the two measurements (*r* = 0.996). However, the algorithm more accurately calculated volumes for the 56 ICH patients without IVH when compared to CTP-based planimetry (*r* = 0.994, *p* < 0.001). Due to the difficulty with calculating HVs with concurrent IVH, few studies have demonstrated algorithms that accurately calculate HVs in these patients (Wang et al., 2021).

Ultimately, the primary limitation of AI and ML algorithms in the current literature is their lack of efficacy in evaluating ICH in the context of IVH or infratentorial hemorrhages, both known to carry a worse prognosis than supratentorial ICH without IVH. Bleeding that extends to the ventricle can be associated with posthemorrhagic hydrocephalus, cerebral palsy, and permanent neurological deficits depending on which neural structures are compressed by the accumulation of ventricular blood (Wang et al., 2021). While the results from Wang et al. demonstrated some promising results, further studies are needed to substantiate the potential for utilizing such algorithms for ICH with concurrent IVH. ML algorithms are currently of limited use in patients with these types of ICH who are invariably more critically ill and, from a neuroanatomical standpoint, have more severe and complex disease. Neuroradiologists often use similar methodologies across each of the studies they read. Therefore, it is imperative to develop algorithms which can be consistently utilized for preoperative planning for any prospective patient with hemorrhagic stroke regardless of the further complications of IVH, hydrocephalus, or infratentorial bleeding.

In clinical practice, neuroradiologists often assess for other predictors of ICH severity on CT scans. For instance, “spot sign,” defined as the presence of more than one focal enhancement within an acute hematoma, is a marker that suggests the presence of ongoing bleeding and often reliably predicts HE (Wada et al., 2007; Hillal et al., 2022). Another radiographic marker of severity is the “satellite sign,” defined as the presence of a visible hemorrhage up to 20 mm away from the original hematoma. Other neuroradiological markers on CT scans assessed for by radiologists and neurosurgeons include “swirl sign,” “black hole sign,” “blend sign,” and “island sign” (Selariu et al., 2012; Hillal et al., 2022). All of these signs are ultimately poor prognostic indicators suggesting that hematoma expansion is likely to occur, but each sign has its own intricacies, sensitivity, and specificity for such predictive values. The current literature has a select few studies which have compared the utilization of CNNs with a pre-existing deep learning model for accurately identifying the above predictive markers (Zhong et al., 2021). One such study was by Zhong et al., which demonstrated that a CNN model could identify “black hole” sign and “blend” sign when compared to a pre-existing deep learning model; however the algorithm failed to consistently identify “swirl” sign (Zhong et al., 2021). Therefore, further studies analyzing the use of such algorithms to consistently identify such radiographic signs are needed. If AI is able to successfully evaluate such radiographic signs with a high degree of accuracy, it could make the clinical training for neuroradiologists and neurosurgeons much more efficient by allowing them to allocate more mental resources to other important tasks needed for patient care.

Overall, results in the current literature have demonstrated the ability of ML algorithms to calculate HVs from CT scans (Kuo et al., 2019; Ma et al., 2022; Peng et al., 2022). A recent 2022 study by Tanioka et al. ventured even further, comparing multiple ML algorithms including XGBoost, random forests, support vector machines, and k-nearest neighbors (k-NN) (Tanioka et al., 2022). The authors suggested that the k-NN algorithm is the most superior given its superior speed and accuracy relative to other algorithms (Tanioka et al., 2022). With improvement in technological advances, institutions are likely to continue developing more ML algorithms with better DSC scores, stronger correlations with gold standard techniques, and faster calculation times.

The utilization of such algorithms provides significant benefits for healthcare teams involved in ICH care. Neuroradiologists who first interpret the film can use the AI to better communicate with neurosurgeons the anatomical and radiographic signs relevant to the patient’s clinical picture. This can ultimately allow for better preoperative planning of the respective neurosurgical intervention, which in the emergency setting, is essential for patients with severe disease. However, further studies are needed to further assess for and substantiate these potential benefits if applied to routine clinical practice.

## 3. Ischemic stroke

### 3.1. Background

Ischemic stroke (IS) is a devastating condition that annually affects nearly 795,000 people in the US alone and 11.6 million people globally (Saini et al., 2021; Tsao et al., 2022). As in ICH, earlier detection of IS can lead to more timely neurosurgical intervention and improvements in long-term morbidity and mortality. Currently, a number of imaging modalities exist that aid in both the detection as well as characterization of ischemic strokes. With the information gleaned from these studies, clinicians can estimate prognosis and make further decisions regarding both medical and surgical management. Significant advancements have been made toward utilizing artificial intelligence (AI) programs to rapidly and accurately identify abnormalities in many of these imaging studies and consequently assist in clinical decision making. The following discussion will explore some of these advancements and the potential role of AI in the diagnosis, prognostication, and management of ischemic stroke.

### 3.2. Detection of acute ischemic stroke

Central to the diagnosis of ischemic stroke is adequate access to accurate and rapid imaging. Although non-contrast computed tomography (NCCT) is often the initial study obtained for suspected stroke cases, its low sensitivity for detecting early ischemic stroke restricts its use primarily to the exclusion of ICH (Chalela et al., 2007). Compared to NCCT (sensitivity: 26%), MRI diffusion-weighted imaging (DWI) remains the most effective modality for the detection of early IS with a sensitivity of at least 83% in most studies (Chalela et al., 2007). Unfortunately, limitations in both accessibility and availability of MRI technology can delay crucial diagnosis. Moreover, a single MRI study takes upwards of 30 min to be performed and at most institutions, with additional delays in time associated with waiting for availability for a scanner in the setting of other emergent indications such as cauda equina syndrome, transporting the patient to and from the machine, and awaiting a confirmed read from an attending radiologist (Puhr-Westerheide et al., 2022). Although MRI is sensitive for IS, studies have shown mixed findings of long-term outcomes for patients with IS who received an MRI as their initial imaging study, which may be related, in part, to delays in imaging and subsequent interpretation (Kleindorfer et al., 2015). Therefore, AI may be of great value for reducing the delays in IS diagnosis seen across US hospitals.

The detection of subtle findings that can otherwise be missed by physicians encompasses the most promising aspects of AI (Nishio et al., 2020; Kaothanthong et al., 2022; Lu et al., 2022). To aid in the detection of “invisible” acute IS, Lu and colleagues developed a deep-learning model comprised of two deep CNNs. The first CNN was a localization model that used pre-labeled NCCT scans with both positive and negative findings to visually outline regions of interest suspicious for infarct. The resulting output was then fed-forward into the second CNN, a classification model that assigned a probability of acute IS to each study. After a training phase consisting of a subset of patients from Tongji Hospital (Institution A), the authors used the remaining patients helped internally validate the model and demonstrated reasonable performance as measured by sensitivity, specificity, accuracy, and AUC, a measure of the predictive power of a model (68.99, 98.22, 89.87, 83.61%, respectively). Even when applied to an external cohort consisting of patient data from The First Affiliated Hospital (Institution B), in which both patient demographics and image acquisition differed from that of Institution A, the model again demonstrated comparable performance in the aforementioned metrics (sensitivity: 62.99%; specificity: 89.65%; accuracy: 88.61%; AUC: 76.32%). This performance was superior to those of two experienced radiologists tested on the same collection of studies (AUC 76.32% vs. 64.01% vs. 64.39%, respectively). Furthermore, the performance of both radiologists improved with the assistance of the model (AUC 81.15 and 81.83%, respectively), suggesting the potential for a synergistic relationship between man and machine.

### 3.3. Characterization of ischemic stroke

After initial detection of IS, clinicians utilize a number of factors to help determine appropriate next steps in management including etiology, time of onset, presence of large vessel occlusion (LVO), core infarct volume, and size of penumbra. Early ischemic imaging findings changes, when present, can be pieced together and quantitatively assessed using the Alberta Stroke Program Early CT Score ASPECT score (Mokin et al., 2017). The evolution of the field of radiomics has allowed an unprecedented degree of speed and accuracy in the extraction of many of these features, especially compared to previously used segmentation methods. The following sections will highlight some of the ways in which machine learning algorithms have been employed to further enhance the characterization of acute ischemic stroke.

#### 3.3.1. Etiology

In 1993, the multicenter Trial of Org in Acute Stroke Treatment (TOAST) clinical trial was completed to develop a classification system of acute IS based on primary etiology (Adams et al., 1993). This yielded five subcategories: large artery atherosclerosis, cardiogenic embolism, small vessel occlusion, IS due to other causes, and IS with unknown cause. Since then, further iterations of the TOAST classification system have been developed to allow for more accurate subtyping; however, the most common causes of IS still belong to the first three subcategories (Wang et al., 2019). Due to the different natures in which these conditions evolve, the treatment strategies for each of these subtypes may also vary. Consequently, accurate and rapid subtyping is an important part of the diagnosis of IS.

To address this, Chen and colleagues developed a deep learning model to categorize causes of embolic IS due to either cardiogenic causes or large artery atherosclerosis (Chen et al., 2023). This model consisted of segmented CTA data that underwent feature extraction using both radiomics and CNN algorithms concurrently. The resulting output was then combined and fed-forward into a subtyping model consisting of nine separate classifiers to determine the most optimal outcome. The authors found the Adaboost algorithm to produce the highest performance (AUC: 0.9018, accuracy: 0.8929), suggesting that such models could potentially assist clinicians in making a timely diagnosis (Chen et al., 2023).

#### 3.3.2. Time of onset

The time of onset since initial symptoms of a stroke is also an important factor to consider when determining treatment options. However, for many patients, this information may not be readily available. In fact, as many as 25% of ischemic strokes occur during sleep, often limiting available options (Lago et al., 1998; Fink et al., 2002; Serena et al., 2003). Previous research by Thomalla and colleagues has shown that diffusion-weighted imaging (DWI) in combination with T2-FLAIR imaging can actually provide insight into the approximate onset of stroke (Thomalla et al., 2011). They showed that the DWI-FLAIR mismatch, if detected, could suggest stroke onset within the prior 4–5 h. However, identifying this mismatch can be a challenging task even for experienced radiologists. Recent work has shown that deep learning could better facilitate this mismatch detection and achieve higher performance in most metrics (as measured by sensitivity, specificity, and accuracy) compared to human readers (Ho et al., 2017; Zhu et al., 2021; Polson et al., 2022).

ML has also shown promise in the field of metabolomics in determining stroke onset. Previous work has been performed to identify possible biomarkers of early stroke with varying degrees of success (Laskowitz et al., 2009; Montaner et al., 2011; Bustamante et al., 2017; Sidorov et al., 2019). Using a metabolomics-based machine learning framework called extreme gradient boost (XGBoost), Zhang and colleagues were able to identify multiple serum biomarkers in rats that were both predictive of the presence of stroke as well as the time of onset (Zhang et al., 2022). Together, these findings show the potential for ML in stroke management beyond the scope of imaging alone. Future areas of research should focus on how AI can be implemented in neurosurgical intervention specifically.

### 3.4. Core infarct volume segmentation

Accurate estimation of this core infarct volume through CTP is essential in guiding further management. However, among available CTP software programs, there is tremendous variability in the parameters used to define core infarct and the resulting perfusion maps (Fahmi et al., 2012; Koopman et al., 2019; Hoving et al., 2022). A number of ML algorithms have been trained using either CTP maps alone or some combination of these perfusion maps with other clinical data (Clerigues et al., 2019; Chen et al., 2020; Wang et al., 2020). The incorporation of these maps introduces inherent variability in the models themselves. Furthermore, each model must first be trained according to the individual CTP software program used prior to clinical application. To bypass these issues, de Vries and colleagues developed a U-Net like model, PerfU-Net, that utilized CTP source data without the use of any intermediate perfusion map (de Vries et al., 2023). The model also utilized the concept of symmetry awareness and skip connections, whereby a potentially infarcted hemisphere was compared to its healthy counterpart and the output from one layer became the input to every other subsequent layer in order to select for particularly salient elements of the image. While PerfU-Net did not perform at the level of some of the top models that did use CTP maps, it demonstrated comparable performance [Dice: 0.46, precision: 0.54, recall: 0.49, average volume difference (AVD) compared to ground truth using MRI-DWI: 12.74]. These findings suggest that AI can help standardize and relatively accurately estimate volumetric measurements of infarct.

### 3.5. Large vessel occlusion

Large vessel occlusion is a prominent cause of acute IS, which carries a disproportionately high level of morbidity and mortality (Smith et al., 2006, 2009; Malhotra et al., 2017). Consequently, rapid detection of LVO is of paramount importance in the initial workup of any stroke. AI has shown tremendous potential in this sphere, leading to the development of commercially available programs, such as and Rapid CTA and *Viz.*ai-LVO, that have reasonably high sensitivity and specificity (Murray et al., 2020; Karamchandani et al., 2023). Some recent work has even demonstrated a role for AI in detecting LVO using CTAs obtained in mobile stroke units (MSUs) (Czap et al., 2022). MSU CTAs exhibit limitations in their capacity to show ischemia in part due to the variability in image quality when compared to traditional scanners as well as an earlier than usual acquisition time after stroke onset. The model developed by Czap and colleagues was initially trained and tested using in-hospital CTAs, achieving an AUC of 0.84. The authors then tested the same model using CTAs obtained in MSUs and achieved a comparable performance with an AUC of 0.80 (Czap et al., 2022). Together, these results show that MSUs equipped with adequately trained machine learning algorithms can assist clinicians in achieving faster diagnoses in the acute setting. The value of this additional speed that AI offers cannot be understated in the acute setting where “time is brain.”

### 3.6. Prognostication of ischemic stroke

In addition to facing the clock, clinicians are often challenged by patients’ families who are concerned regarding the acute and long-term prognosis following their stroke. The answer to this question may vary depending on a number of factors including clinical features, radiological findings, and medical history as well as the treatment options available on presentation. The Alberta Stroke Program Computed Tomography Score (ASPECTS) was one of the first efforts to predict outcome following an MCA stroke as it allowed for the quantification of ischemic changes in the anterior circulation using NCCT (Barber et al., 2000). It was originally designed to identify those patients that would benefit from thrombolytic therapy. Since then, it has been used to decide which patients may benefit from mechanical thrombectomy (Yoo et al., 2014; Goyal et al., 2015; Powers et al., 2015).

Initially, the manual interpretation of ASPECTS was tedious and often showed variability due to a number of factors including human rater experience (Wardlaw et al., 2007; Menon et al., 2011). Today, the advent of commercially available AI software such as RapidAI’s Rapid ASPECTS and Brainomix’s e-ASPECTS has automated this process, resulting in improved inter-rater agreement (Nagel et al., 2017; Goebel et al., 2018; Shibata et al., 2022; Chan et al., 2023). Additionally, several studies have produced models that have been able to generate ASPECTS scores from NCCT in high concordance with ASPECTS scores derived from DWI (Albers et al., 2019; Kuang et al., 2019). These performances were superior to that achieved by experienced clinicians, thus suggesting that AI can play a future role in ischemic stroke management and not just diagnosis.

In addition to these programs, there are other ML models that have been developed to predict outcomes following IS. For example, AI has been used to predict the occurrence of specific events immediately following IS including malignant cerebral edema as well as hemorrhagic transformation and reinfarction following thrombectomy (Yu et al., 2018; Foroushani et al., 2020; Choi et al., 2021; Hoffman et al., 2023). Such measures may allow future clinicians to determine disease severity earlier in the management of IS patients. Multiple studies have demonstrated efforts to develop ML algorithms that can predict functional outcomes at various stages post-discharge (Forkert et al., 2015; Dragoș et al., 2023; Ozkara et al., 2023). Although the success of these studies is somewhat limited, it shows great promise for the future of IS care.

### 3.7. AI software implemented in practice in cerebrovascular accident patients

Through the iterative enhancements of AI demonstrated in the literature outlined above for CVA patients, there has been the recent implementation of commercial software platforms at various stroke centers utilizing AI for both ischemic and hemorrhagic stroke, some of which are highlighted in Table 1.

**Table 1:** Commercially utilized software applications utilizing AI in ischemic stroke and ICH.

Software	Application	Clinical use	Imaging modality
RapidAI	Rapid ICH	Detection and classification of ICH	CT
	Rapid CTA	Detection of LVO	CTA
	Rapid CTP	Detection of perfusion mismatch	CTP
	Rapid MR	Detection of perfusion mismatch	MRI/MRA
	RapidASPECTS	Facilitates ASPECTS grading for stroke	CT
Brainomix	e-ASPECTS	Facilitates ASPECTS grading for stroke	CT
	e-Blood	Detection and quantification of ICH	CT
	e-CTA	Detection of LVO, collateralization	CTA
	e-Mismatch	Detection of perfusion mismatch	CTA/CTP/MRA
*Viz.*ai	*Viz* LVO	Detection and triage of LVO	CTA
	*Viz* CTP	Detection of perfusion mismatch	CTP
	*Viz* ICH	Detection and triage of ICH	CT
Aidoc	Aidoc LVO	Detection and triage of LVO	CTA
	Aidoc CTP	Detection of perfusion mismatch	CTP
	Aidoc ICH	Detection and triage of ICH	CT
Avicenna.AI	CINA LVO	Detection and triage of LVO	CTA
	CINA ASPECTS	Facilitates ASPECTS grading for stroke	CT
	CINA ICH	Detection and triage of ICH	CT

**Figure 3:**
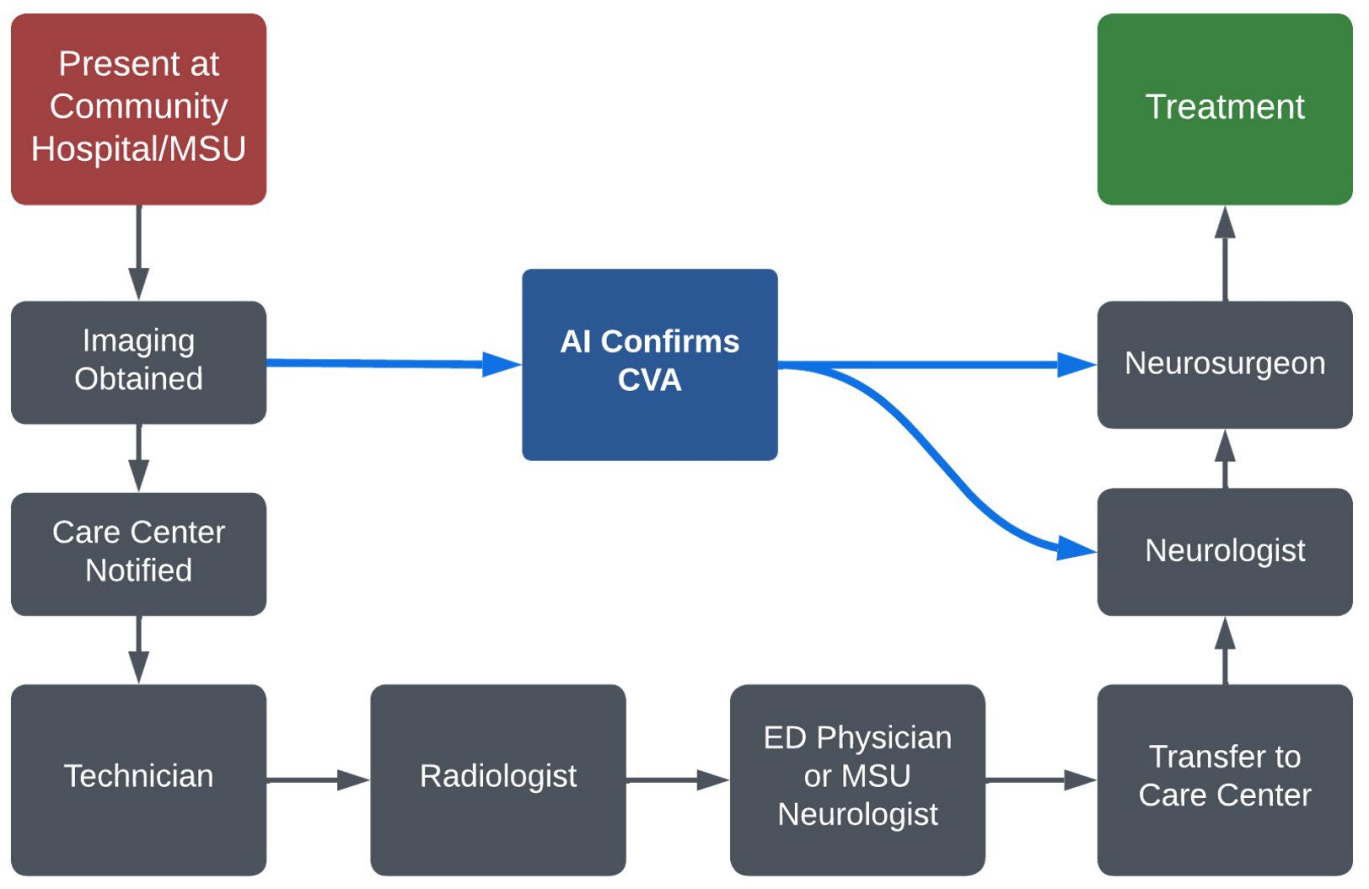
The use of AI software from an outside hospital to a major stroke center for a cerebrovascular patient requiring complex stroke intervention.

These software applications facilitate a systematic approach to suspected CVA patients, and can expedite the process of detecting CVA, and triaging who is eligible and may benefit from neurosurgical intervention. The practical implementation of AI software from a community hospital to a major stroke center is outlined in Figure 3.

## 4. AI in intracranial aneurysms

Intracranial aneurysms (IAs) have a prevalence of approximately 3.2% in the general adult population. While most aneurysms are found incidentally, there is always a risk of rupture that is associated with a 25% mortality rate during the first 24 h. Ruptured aneurysms are responsible for approximately 500,000 annual deaths worldwide, with the risk of rupture increased with larger aneurysms (Rinkel et al., 1998; Vlak et al., 2011; Morita et al., 2012; Jersey and Foster, 2023). Unfortunately, most patients with cerebral aneurysms are unaware of their condition since they remain asymptomatic until a rupture occurs and leads to long-term neurological deficits. Therefore, the accurate and timely diagnosis of aneurysms is essential for astute clinical decision-making regarding intervention and subsequent clinical outcomes. With respect to unruptured aneurysms, there is still debate on the specific guidelines regarding if and under what circumstances intervention is warranted, the prognostication of aneurysms, as well as the preferred form of neurosurgical intervention (Thompson et al., 2015). The introduction of AI, specifically DL algorithms, shows promise for improving present systems for the detection and prognostication of aneurysms.

### 4.1. The role of AI in aneurysm detection

Imaging modalities typically used for detection of aneurysms are similar to those used in the acute ICH setting, and they include DSA, CTA, and MRA. Each of these modalities can be operator-dependent and has varying degrees of accuracy in detecting subtle arterial lesions. Some of the potential diagnostic inaccuracies have been mitigated through the implementation of computer-aided diagnosis (CAD) systems. These are automated systems which, through the use of algorithms, can assist in the detection of abnormal imaging findings through the analysis of certain imaging features such as arterial wall defects (Arimura et al., 2004; Lauric et al., 2010; Yang et al., 2011; Hanaoka et al., 2019). While much of the earlier literature cited a high false positive rate of CAD, the recent incorporation of DL models, have allowed for greater adaptations and potential improvements to such systems. In theory, efficient DL models can facilitate greater sensitivity of this system in detecting IAs across various forms of imaging. AI can additionally process the dynamic nature of aneurysms to better understand its true size and morphology than simple diameter measurement can provide (Sahlein et al., 2022).

DSA is currently recommended as the first-line imaging modality due to its high sensitivity in detecting aneurysms of all sizes (Thompson et al., 2015). When utilized with AI, CNN classifiers have yielded promising results in retrospective studies, with the literature citing a sensitivity of 79–100% (Jerman et al., 2017; Duan et al., 2019; Hainc et al., 2020; Jin et al., 2020; Zeng et al., 2020; Ou et al., 2022). These studies have analyzed a wide array of algorithms, such as RAGS, RetinaNet, YOLOv3, ViDi, and UNet. Jerman et al. demonstrated a 100% sensitivity using a 7-layer 2D-CNN model (Jerman et al., 2017). Hainc et al. utilized a commercially available ML system on 2D images, and even while demonstrating a comparatively lower sensitivity (79%) of this algorithm, they demonstrated the feasibility of using AI, with the capacity for further improvement with the utilization of 3D images and manual ML training (Hainc et al., 2020). This can lead to lower error rates and earlier detection times in asymptomatic patients who are unbeknownst to their own risk of aneurysm rupture.

CTA is a less invasive imaging modality to diagnose aneurysms with a similarly high sensitivity and specificity rate. Its main limitations are the susceptibility to artifact and decreased sensitivity for smaller aneurysms compared to DSA (Thompson et al., 2015). Often, aneurysm detection is enhanced by vasculature rendering and bone subtraction. As a result, the utilization of ML in combination with CTA has a sensitivity that differs based on aneurysm characteristics, with many studies utilizing the HeadXNet model, UNet, and DeepMedic algorithms (Park et al., 2019; Shahzad et al., 2020; Shi et al., 2020; Alwalid et al., 2021; Bo et al., 2021; Pennig et al., 2021; Yang et al., 2021; Liu et al., 2023). Compared to other radiographic combinations, CTA had the most widespread sensitivities with high dependence on size and location. This was demonstrated by Shi et al., who analyzed the DaResUNet model, and detected a sensitivity ranging from 51.7 to 100% and from 60.6 to 100% for location (Shi et al., 2020). In spite of these limitations, other studies have demonstrated the overall efficacy of augmentation AI algorithms with clinicians and reliable segmentation (Park et al., 2019). The FDA-approved *Viz.*AI Aneurysm CNN had a sensitivity of 93.8% and accuracy of 94.0% for aneurysms in their approved range of 4 mm or larger with a mean processing time of 114.7 s (Colasurdo et al., 2023). RAPID Aneurysm was one of the most promising accurate programs, demonstrating a sensitivity of 95% and accuracy of 99% across 51 patients (Heit et al., 2022). However, there remains a dearth of available data pertaining to CTA with ML in aneurysm diagnosis and management.

With respect to MRA, when using ML techniques with CAD, the sensitivities for aneurysm detection ranged from 70 to 100%, depending on technique and size (Nakao et al., 2018; Sichtermann et al., 2019; Ueda et al., 2019; Chen et al., 2020, 2023; Faron et al., 2020; Joo et al., 2020; Shimada et al., 2020; Sohn et al., 2021; Terasaki et al., 2021). Most of these models utilized validated ML algorithms UNet, DeepMedic, and ResNet with retrospective data. These include the 2D models of MRA and TOF-MRA, with higher sensitivities found for larger aneurysms and when the model was used in concert with human readers (Nakao et al., 2018; Sichtermann et al., 2019; Stember et al., 2019; Faron et al., 2020). Nakao et al. employed a “2.5D” approach by having the network input 2D-image representations of 3D objects through the combination of a CNN and MIP algorithm created to be incorporated with pre-existing CAD systems. This demonstrated similar outcomes to 3D network models (Chen et al., 2020; Faron et al., 2020; Joo et al., 2020; Sohn et al., 2021). Chen et al., using UNet, noted significant decreases in false positives (0.86 per case) compared to most other studies when running the algorithm after use of an automated vessel segmentation algorithm (Chen et al., 2020). The same authors conducted a 2023 multi-center follow-up study, in which they improved their Squeeze-and-Excitation (SE) 3D UNet network optimized with a five-fold cross-validation, which yielded an increase in 15.79% for patient-level sensitivity and further decrease in false positives (Chen et al., 2023). ML with CAD may be a suitable option for aneurysm detection in clinical scenarios where patient care is satisfied by noninvasive imaging alone.

Terasaki et al. have conducted one of the few multi-center studies evaluating ML models of 2D-CNN, 3D-CNN, and multidimensional CNN using TOF-MRA images with both 3.0 and 1.5T (Terasaki et al., 2021). The authors found the highest sensitivities rates of 82.1, 86.5, and 89.1% in the external tests with false positive rates of 5.9, 7.4, and 4.2%, respectively, validating this approach of AI and ML with MRA imaging (Terasaki et al., 2021) Of note, this study also displays the overall trend of high false positive rates of MRA compared to CTA and DSA. Teresaki et al.’s high false positive rate may lead to unnecessarily aggressive management being taken by clinicians if they solely rely on the AI to make their diagnosis. This can lead to a waste of scarce and expensive resources in the acute setting and may limit the trust that clinicians have in AI when managing patients with unruptured IAs. This is in stark contrast to Chen et al.’s findings whereby demonstrating fewer false positive rates may allow physicians to better trust these algorithms for guiding management. Future research on AI should attempt to mitigate the false positive rate as low as possible in order to allow for a more feasible AI implementation into the acute setting.

### 4.2. The role of AI in determining aneurysm rupture risk and prognostication

Upon detection of an unruptured IA, the next step is to determine whether surgical intervention is indicated. While no clear consensus hitherto exists, there are scoring systems such as the PHASES score, Ruptured Resemblance Score (RRS), and ELAPSS score which can guide clinical judgment (Greving et al., 2014; Thompson et al., 2015; Backes et al., 2017; Rajabzadeh-Oghaz et al., 2020). The PHASES score provides risks of IA rupture based on the following: population ethnicity, hypertension, age, size of aneurysm, earlier subarachnoid hemorrhage (SAH) from another IA, and site of IA. ELAPSS analyzes the location of the IA, patient age, size, and shape of the IA, and earlier SAH. The RRS assesses the similarity of a current IA to a cohort of previously ruptured IAs based on hemodynamic-morphological parameters such as wall shear stress. Current guidelines suggest that the greatest factors to consider for potential intervention are age, aneurysm location, aneurysm size, type of treatment center, and the available specialized intraoperative tools and techniques, such as intraoperative angiography. Non-sphericity index scores are evaluated through intraoperative angiography to objectively assess aneurysm characteristics, with higher scores correlating with more irregular shapes. The current literature shows that future growing aneurysms generally exhibited higher NSI scores (Hotta et al., 2014; Dhar et al., 2020).

One goal of ML in this setting is to predict the risk of rupture with incidental aneurysms. Various studies have demonstrated higher degrees of accuracy in predicting IA rupture with ML, particularly when compared to models using limited morphological features or the aforementioned scoring systems (Liu et al., 2018, 2019; Zhu et al., 2020; Bizjak et al., 2021; Sahlein et al., 2022; Li et al., 2023). Rapid Aneurysm, a newer tool from the RapidAI developers that have already implemented several tools into clinical practice, was recently used in a retrospective review of ruptured aneurysms that were treated conservatively (Sahlein et al., 2022). The authors found relative volumetric minimum enlargements of 6%, that were initially deemed undetectable by manual linear measurement (Sahlein et al., 2022).

Bizjak et al. demonstrated the use of models in predicting IA growth based on morphological features. The authors obtained morphological data for 44 IAs from CTA and MRA that subsequently utilized “deep shape” learning via the PointNet++ model to extract vascular surface meshes from the images to predict future aneurysm growth and rupture. This study demonstrated high sensitivity (0.96) and satisfactory accuracy (0.82) (Bizjak et al., 2021).

Furthermore, ML may also be particularly useful for smaller-sized aneurysms (≤7 mm in diameter), for which the risk of rupture or growth is often harder for clinicians to predict (Ahn et al., 2021; Lee et al., 2021; Xiong et al., 2022). This has been demonstrated in a recent systematic review using meta-regression that found no significant predictors for small aneurysm growth or size (Lee et al., 2021). Therefore, the AI/ML granularity for aneurysm growth in this population is of particular note. Xiong et al. created an ML model in 1,400 patients in conjunction with a support vector machine (SVM) algorithm which utilizes supervised learning models with associated learning algorithms to analyze data for classification and regression analysis. The authors noted that SVM outperformed the PHASES score in predicting aneurysm rupture with an AUC of 0.817 and 0.893 in the internal and external validity cohorts, respectively. Through the use of ML, the authors concluded that maximum size, location, and irregular shape of the IAs were the major predictors of aneurysmal rupture (Xiong et al., 2022).

**Figure 4:**
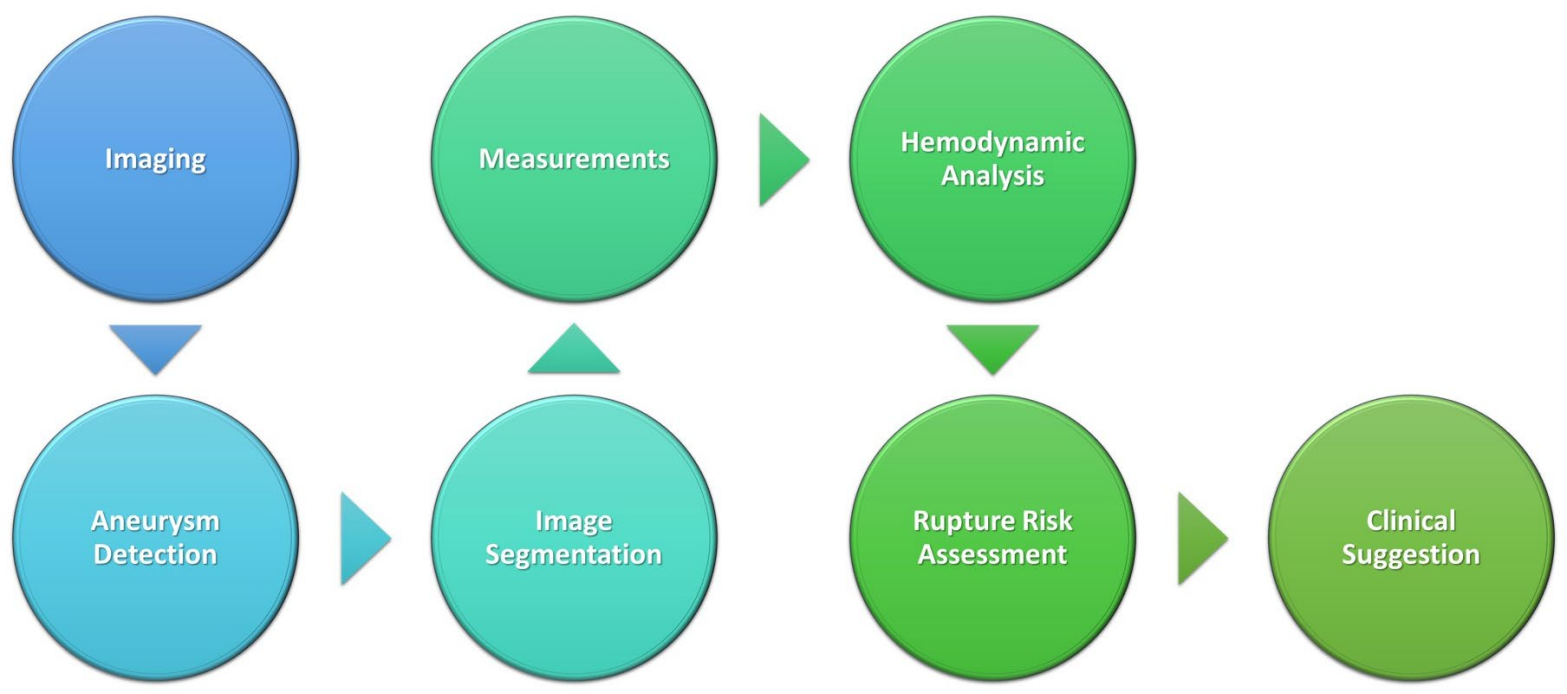
Schematic of AI process in aneurysm detection and clinical decision-making.

The highest predictive value for rupture risk in aneurysm patients was observed when algorithms include data beyond imaging characteristics, particularly when adding hemodynamic characteristics and clinical information (Chen et al., 2020, 2022; Detmer et al., 2020; Ou et al., 2022). Chen et al. was one of the first researchers to utilize hemodynamic characteristics in their modeling, finding them to be a more significant predictor than imaging in their retrospective study with 1,007 IA patients (Chen et al., 2020). Their tested algorithms (including random forest modeling, multilayer perceptron, and SVM) trained on two different hospital sets performed similarly to traditional logistic regression modeling. In 2022, researchers at Capital Medical University similarly compared different ML and deep learning algorithms both with and without hemodynamic features, noting greater accuracy for almost every measure when hemodynamic features were included (Chen et al., 2022). An example of this workflow is shown in Figure 4.

### 4.3. AI in predicting outcomes after IA intervention

ML has also been utilized not just in predicting clinical outcomes on presentation, but also in predicting the risk of occlusion after interventions with various endovascular devices. Various ML systems such as ElasticNet and UNet have been utilized in this way yielding sensitivity results ranging from 75 to 98% (Paliwal et al., 2018; Shiraz Bhurwani et al., 2020; Guédon et al., 2021; Jadhav et al., 2022). These studies generally utilized DSA imaging, but spanned multiple interventional methods including flow diverters, intrasaccular embolization devices, and pipeline embolization devices.

Paliwal et al. retrospectively analyzed 84 ICA sidewall aneurysms treated with flow diverters with ML algorithm parameters, including factors such as hemodynamics, morphology metrics, and morphometrics neck ratio (calculated on the 2D DSA images) (Paliwal et al., 2018). The most sensitive ML algorithm tested was the Guassian-SVM and Neural Network (with 90% accuracy). However, all 5 algorithms used in this study demonstrated significant improvement when using the “all-parameters” model that included 16 inputs compared to the” significant parameters” model which only included five inputs. Guedon et al. also studied ML algorithms in the context of flow diverters with the aim of utilizing AI to address the lack of scoring criteria for occlusion prediction after this treatment. The authors included 146 subjects with ElasticNet for feature selection and outcome prediction (occlusion or no occlusion). They demonstrated their DIANES score to have an 89% sensitivity and 81% accuracy (Guédon et al., 2021). Additionally, Guedon et al. and Paliwal et al. found that neck ratio may be a significant factor in occlusion 6 months after treatment (Paliwal et al., 2018; Guédon et al., 2021).

Jadhav et al. specifically focused on wide-neck bifurcation aneurysms treated with an intrasaccular device defining occlusion outcomes according to the Raymod-Roy Occlusion Classifications, which classifies aneurysm occlusions into three categories (complete occlusion, residual neck, and residual aneurysm), rather than the binary outcomes hither to reported in prior literature (Jadhav et al., 2022). The authors then created different “feature sets” that each contained different input combinations and analyzed each set with different algorithms. Random forest modeling used with a feature set combining clinical and imaging features displayed the highest accuracy of 75.3% and sensitivity of 91.8%. The authors additionally developed a neural network segmentation algorithm similar to UNet to automate 2D and 3D image characteristic calculations which performed similarly to the manual computations.

The long-term outcomes of patients with IAs is an important topic that can be addressed by advancing technology as well. Bhurwani et al.’s research defined outcomes as occlusion or no occlusion at 6 months offering predictive postoperative values for patients treated with pipeline embolization devices (Shiraz Bhurwani et al., 2020). They utilized Keras with DSA imaging and angiographic parametric imaging to train a deep neural network, finding an average sensitivity of 0.92 but a specificity of only 0.57. Other studies found higher predictive values when separating full occlusions from partial occlusions, which may account for the wide range in sensitivities within the literature (Jadhav et al., 2022). The combination of imaging and clinical factors improves sensitivity as well, with Guedon et al. utilizing ElasticNet to create the DIANES grading scale with an 89% sensitivity and 81% accuracy (Guédon et al., 2021). Further investigations are needed to reach a clearer consensus on the effectiveness of ML in predicting occlusion outcomes.

Of note, a novel ML algorithm was presented in 2021 by Williams et al., with their proposal of the Aneurysm Occlusion Assistant to provide real time surgical guidance (Williams et al., 2021). Using the open source softwares Keras, Tensorflow, and skLearn combined with angiographic parametric imaging and segmented DSA imaging, the authors demonstrated predictions on occlusion risk after device placement within 7 s, with reasonable prediction accuracy for occlusion at 6-months (0.84) This avenue of prognostication yields significant potential for cerebrovascular neurosurgeons and requires further investigation.

## 5. Arteriovenous malformations

AVMs are aberrant, dysfunctional connections between arteries and veins without an intervening capillary bed (Cockroft et al., 2012). These aberrant lesions consist of an intervening nidus with an intertwining of blood vessels that have a high propensity to bleed (Cockroft et al., 2012). Brain AVMs frequently present with rupture which can lead to loss of consciousness, permanent neurological deficits, and death. Non-ruptured AVMs can be detected incidentally, or present with headaches, progressive neurological deficit, or seizure.

### 5.1. Current literature on the role of AI in AVMs

AVMs appear to have a greater propensity to bleed in pediatric patients compared to adults (El-Ghanem et al., 2016). Saggi et al. utilized three ML algorithms, random forest models, gradient boosted decision trees, to predict the risk of hemorrhage for AVMs in 189 pediatric patients who presented with or without hemorrhage (Saggi et al., 2022). The ML algorithm discerned that smaller AVM sizes, left-sided AVMs, and the presence of a concurrent arterial aneurysm were all predictors of hemorrhage on presentation. When compared with a conventional regression approach, only the ML algorithm was able to pick up on these subtleties.

Few studies have demonstrated that AI can successfully predict hemorrhage secondary to AVM in adult patients. Across 1810 patients, Oermann et al. developed a 3D-surface ML algorithm model that accurately predicted the risk of adverse events in patients who received radiosurgery for AVM solely based off imaging findings (Oermann et al., 2016). Oermann et al. compared their algorithm to widely used scoring systems such as Spetzler-Martin grading scale (Spetzler and Martin, 1986), radiosurgery-based AVM score (RBAS) (Pollock and Flickinger, 2002), and the Virginia Radiosurgery AVM Scale (VRAS) (Starke et al., 2013). For all three comparisons of the algorithm, the AUC for the predictive capability ranged from 0.6 to 0.7 depending on the time point at which the comparison was made. Jiao et al. performed a similar study where AI-based indicators were used to predict the likelihood of postoperative motor deficits in patients who received AVM resection surgery (Jiao et al., 2023). When compared to the Spetzler-Martin grading scale, the highest AUC was observed in the logistic regression model of 0.88 (Jiao et al., 2023).

### 5.2. Limitations and future directions for ML algorithms in cerebrovascular neurosurgery

Although novel machine learning (ML) techniques show significant promise for cerebrovascular surgery, there are still many limitations that must be accounted for prior to further application in clinical practice. Of note, the majority of the studies analyzing ischemic stroke, ICH, AVM, and aneurysms were retrospective in nature with small sample sizes. Furthermore, much of the literature utilizes retrospective analyses, meaning that the actual, prospective implementation of these models is still pending. These mostly single center studies lead to small datasets on which the ML algorithms can train. The reported sensitivities and accuracies for these models are thus difficult to compare and generalize. Additionally, significant manual input and calculation was required to analyze the desired parameters. This minimizes the automaticity and time saving potential of AI as a clinical tool. There is a greater need for multi-center prospective studies to substantiate such AI algorithms prior to their full implementation into clinical practice. The lack of substantive studies evaluating AI is specifically notable for vascular lesions such as aneurysms and AVMs.

Moreover, when deep learning models are developed, they take into consideration how the institution’s electronic medical record (EMR) system and picture archiving/communication systems (PACS) will display radiological films. A major drawback that applies to most of the studies noted in this review is that they are single-center studies. Therefore, it would be difficult to implement any of these ML algorithms at other institutions where the PACS software and CT imaging protocols for ICH patients may have subtle differences. Many critically ill ICH patients at larger academic centers are often transferred from smaller outside hospitals where the initial head CT was performed (Vahidy et al., 2016). A successful algorithm is one that can be implemented at both tertiary centers and smaller hospitals with differing software. Future studies should consider the flexibility of ML algorithms so that they can evaluate the algorithms’ efficacy in samples across multiple institutions with different EMRs and PACS software.

There is also the ethical dilemma that comes with the use of an automated system in clinical decision making for cerebrovascular neurosurgeons. AI can be beneficial in creating more standardization between physician detection and decisions while also analyzing more variables or in identifying more minute vascular abnormalities the human eye can. However, the large rates of false positives shown in the literature are proof of principle which is two-fold. Firstly, of the need for further investigation and iterative improvement in these algorithms, and secondly, that these algorithms are a useful adjunct but are no substitute for the judgment of a clinician.

Nevertheless, AI provides a promising avenue to revolutionize the practice of modern neurosurgery, and further lines of inquiry are needed both to improve on what has been done, and to open up further avenues of use in cerebrovascular surgery.

## 6. Conclusion

Cerebrovascular disorders often carry a significant management morbidity and mortality, making timely diagnosis and intervention essential. The application of AI, particularly ML and DL, in the realm of cerebrovascular neurosurgery has facilitated a more expedited detection, triaging, and prognostication of cerebrovascular pathologies such as ischemic strokes, ICH, AVMs, and aneurysms. Moreover, the literature has demonstrated that having this technology has conferred improvements in timely detection of these pathologies for physicians in training. Some of these AI software applications have already been implemented into clinical practice, particularly in the realms of ICH and ischemic stroke. As this technology continues to develop and be applied in increasingly innovative ways, it is important to remember that many of these models depend on a pre-determined gold standard to determine ground truth. As such, their maximum potential may be limited by the information immediately available to the algorithm. Clinicians must ultimately go beyond simply evaluating how certain machinery may be beneficial in the pre, intra, or post-operative setting. In order to ensure patient safety, physicians and their staff should have a thorough understanding of the methodology used to develop AI, its advantages and limitations in the clinical setting as well as the barriers to its implementation for specific patient populations. Future multicenter prospective studies are needed to further substantiate these algorithms for further application in clinical practice, particularly with vascular lesions such as aneurysms and AVMs. While in its relative nascency in clinical practice, AI technology provides significant promise as an adjunct for neurosurgeons in revolutionizing clinical decision-making and subsequent clinical outcomes in cerebrovascular surgery.

## Author contributions

RD: Conceptualization, Methodology, Supervision, Writing – original draft, Writing – review & editing. KG: Conceptualization, Methodology, Writing – original draft. SS: Conceptualization, Methodology, Writing – original draft. RM: Conceptualization, Writing – original draft, Writing – review & editing. JB: Conceptualization, Methodology, Writing – original draft.

## Conflict of interest

The authors declare that the research was conducted in the absence of any commercial or financial relationships that could be construed as a potential conflict of interest.

## Publisher’s note

All claims expressed in this article are solely those of the authors and do not necessarily represent those of their affiliated organizations, or those of the publisher, the editors and the reviewers. Any product that may be evaluated in this article, or claim that may be made by its manufacturer, is not guaranteed or endorsed by the publisher.

## Figures and Tables

**Table:** 

AI	Artificial intelligence
ML	Machine learning
DL	Deep learning
AVM	Arteriovenous malformation.
ICH	Intracerebral hemorrhage
IA	Intracranial aneurysm
CVA	Cerebrovascular accident
NCCT	Non-contrast computed tomography
ANN	Artificial neural network
CNN	Convolutional neural network
RNN	Recurrent neural network
IVH	Intraventricular hemorrhage
SAH	Subarachnoid hemorrhage
SDH	Subdural hematoma
EDH	Epidural hematoma
IPH	Intraparenchymal hemorrhage
CTA	Computed tomography angiogram
CTA	Computed tomography perfusion
DSA	Digital subtraction angiography
MRI	Magnetic resonance imaging
HE	Hematoma expansion
PHE	Perihematomal edema
SVM	Support vector machine
k-NN	k-nearest neighbor
DSC	Dice similarity coefficient
AVD	Average volume difference
AUC	Area under the curve
DWI	Diffusion-weighted imaging
TOAST	Trial of Org in Acute Stroke Treatment
FLAIR	Fluid-attenuated inversion recovery
ASPECTS	Alberta Stroke Program Computed Tomography Score
TOF	Time-of-flight
SE	Squeeze-and-excitation
RBAS	Radiosurgery-based AVM Score
VRAS	Virginia Radiosurgery AVM Scale
EMR	Electronic medical record
PACS	Picture archiving/communication systems
